# High Insulin and Leptin Increase Resistin and Inflammatory Cytokine Production from Human Mononuclear Cells

**DOI:** 10.1155/2013/487081

**Published:** 2012-12-24

**Authors:** Panayoula C. Tsiotra, Eleni Boutati, George Dimitriadis, Sotirios A. Raptis

**Affiliations:** ^1^Hellenic National Center for the Research, Prevention and Treatment of Diabetes Mellitus and its Complications (H.N.D.C), 3 Ploutarchou, 10675 Athens, Greece; ^2^2nd Department of Internal Medicine, Research Institute and Diabetes Center, Athens University Medical School, University General Hospital “Attikon”, 1 Rimini, Athens, 12462 Haidari, Greece

## Abstract

Resistin and the proinflammatory cytokines, such as TNF-**α**, IL-6, and IL-1**β**, produced by adipocytes, and macrophages, are considered to be important modulators of chronic inflammation contributing to the development of obesity and atherosclerosis. Human monocyte-enriched mononuclear cells, from ten healthy individuals, were exposed to high concentrations of insulin, leptin, and glucose (alone or in combination) for 24 hours *in vitro*. Resistin, TNF-**α**, IL-6, and IL-1**β** production was examined and compared to that in untreated cells. High insulin and leptin concentrations significantly upregulated resistin and the cytokines. The subsequent addition of high glucose significantly upregulated resistin and TNF-**α** mRNA and protein secretion, while it did not have any effect on IL-6 or IL-1**β** production. By comparison, exposure to dexamethasone reduced TNF-**α**, IL-6, and IL-1**β** production, while at this time point it increased resistin protein secretion. These data suggest that the expression of resistin, TNF-**α**, IL-6, and IL-1**β** from human mononuclear cells, might be enhanced by the hyperinsulinemia and hyperleptinemia and possibly by the hyperglycemia in metabolic diseases as obesity, type 2 diabetes, and atherosclerosis. Therefore, the above increased production may contribute to detrimental effects of their increased adipocyte-derived circulating levels on systemic inflammation, insulin sensitivity, and endothelial function of these patients.

## 1. Introduction

Adipose tissue secretes a number of bioactive molecules, such as resistin, TNF-*α*, and IL-6 collectively called adipokines, which affect peripheral insulin sensitivity and may be important players in the development of type 2 diabetes and atherosclerosis [1, 2]. On the other hand, these molecules expressed by the human macrophages can modulate chronic inflammation and have an impact in cardiometabolic complications such as atherosclerosis. Increased production of these adipokines occurs with expanding obesity, particularly visceral obesity, by both the adipocytes and the nonfat cells, mostly macrophages that infiltrate the adipose tissue [3].

 The adipokine, resistin, was proposed originally to be a link between obesity and type 2 diabetes [4]. Administration of recombinant resistin in rodents impaired hepatic insulin sensitivity and glucose metabolism [5, 6], and treatment with anti-resistin anti-sense oligonucleotides reversed hepatic insulin resistance [7]. Although in humans there were studies demonstrating increased serum resistin levels in individuals with obesity and/or type 2 diabetes [8–10], resistin's biological role as an insulin resistance molecule was debated by others, which failed to confirm a causal relationship of circulating resistin with insulin sensitivity or other metabolic parameters [11, 12]. Notably, in mice resistin is produced solely by the adipose tissue, whereas in humans resistin is barely detectable in adipocytes, and its mRNA levels are much higher in monocytes and macrophages [13, 14]. Along with this, resistin's proinflammatory nature in man was suggested by the finding that human resistin could stimulate the expression of the proinflammatory cytokines TNF-*α* and IL-6 in both human and murine macrophages via the NF-kB-dependent pathway [15, 16], while intravenous administration of endotoxin and activation of this inflammatory cascade could result in hyperresistinemia in humans [17], indicating the importance of this signalling pathway in the resistin-mediated inflammation [15]. In agreement with these experimental data, serum resistin concentrations are significantly elevated in patients with severe inflammatory disease [18] and correlate with markers of inflammation in their inflamed joints [16]. Resistin has also been shown to have direct effects on endothelial cell activation by inducing the expression of endothelin-1 (ET-1) and the cell adhesion molecules VCAM-1 and ICAM-1 [19, 20], and resistin's secretion by the atherosclerotic macrophages could promote atherosclerosis in humans [21]. Indeed, circulating resistin is significantly increased in patients with previous myocardial infarction [22], while it is positively correlated with markers of inflammation in patients with coronary artery disease (CAD) [23] and the risk of heart failure in the Framingham Offspring study [24]. 

Proinflammatory cytokines, such as TNF-*α*, IL-6, and IL-1*β*, circulating and locally produced in the endothelium, by tissue macrophages are thought to be involved in the atherogenic process. They have been shown to induce the expression of cellular adhesion molecules in the endothelium, thus, facilitating the entrapment of leukocytes and monocytes and the initiation of the atherogenic process [25]. Proinflammatory cytokines also increase fibrinogen production and enhance thrombosis, thus increasing the risk of vascular occlusion and cardiovascular events. Thus, elevated TNF-*α* plasma levels are associated with abdominal obesity and an increased risk of myocardial infraction in men [26, 27], while IL-6 circulating levels increase with obesity and are associated with increased risk for myocardial infarction and new onset type 2 diabetes [28, 29].

In the present study, we examined the *in vitro* effects of 24 h exposure to high concentrations of insulin, leptin, and glucose, commonly seen in obesity and type 2 diabetes, on the mRNA expression and protein secretion of resistin, TNF-*α*, IL-6, and IL-1*β* from human peripheral blood monocyte-enriched mononuclear cells. We also compared these effects to those of dexamethasone, a potent anti-inflammatory agent and correlated them to measures of obesity and insulin resistance. 

## 2. Methods

### 2.1. Subjects

Ten healthy volunteers, aged 31 to 41 years, (BMI: 25.7 ± 1.2), participated in our study. The study was approved by the Hospital Ethics Committee in accordance with the Declaration of Helsinki, and all volunteers gave written informed consent. All subjects had no history of recent infection, myocardial infection, or CAD, and were taking no medication. Body Mass Index (BMI) was calculated as the ratio of weight (Kg) per height in the square (m^2^). Insulin resistance was calculated by the homeostasis model assessment index (HOMA-IR) as (fasting insulin (IU/L) × fasting glucose (mmol/L))/22.5. Fasting glucose (YSI 2300 STAT Plus Glucose & Lactate Analyser, YSI Incorporated, Ohaio, USA), insulin levels (INSI-CTK irma, DiaSorin, Saluggia, Italy), and free fatty acid (FFA) levels (Falcor 300 Chemical Analyser, Menarini Diagnostics, Italy) were measured in the plasma from all individuals. High sensitivity C-reactive protein (CRP) was measured using the BN Prospec Nephelometer (Dade Behring, Newark, DE, USA) according to manufacturer's instructions. 

### 2.2. Mononuclear Cell Isolation and Incubation Protocol

Peripheral blood was obtained from all subjects after an overnight fast and the mononuclear cells were separated from heparinized blood samples immediately after collection, using a Ficoll-Paque (Amersham, GE Healthcare Bio-Sciences AB, Uppsala, Sweden) gradient [30], while the plasma from the same samples was separated, aliquoted, immediately frozen, and kept at −80°C until assayed.

The isolated mononuclear cells were left selectively to adhere into a 100 mm-petri dish for 1 hour for the isolation of the primary monocyte-derived macrophages, as it has been described previously [31]. The adhered monocyte-enriched mononuclear cells were collected, counted, and plated at a density of 0.8 × 10^6^ cells/well in 24-well plates in RPMI 1640 supplemented with 10% Fetal Bovine Serum, 2 mM L-glutamine, and penicillin/streptomycin under an atmosphere of 5% CO_2_ at 37°C. Cells were left to incubate with either of the following agents: 138.9 nM insulin (Humulin Regular, Lilly France, Fegersheim, France), 100 ng/mL human leptin (R&D Systems, Oxon, UK), 33 mM glucose (GIBCOBRL, Invitrogen Corp., Carlsbad, USA), 10^−7 ^M dexamethasone (G. A. Pharmaceuticals, Athens, Greece), or combination of the former three agents for 24 hrs. After the incubation period, the medium was collected for measurement of the resistin, TNF-*α*, IL-6, and IL-1*β* secreted proteins, while at the same time total RNA was extracted from the cell pellet. Cell viability and cell counting were done using the trypan blue exclusion.

### 2.3. RNA Extraction and cDNA Synthesis

 Total RNA was isolated from the human peripheral monocyte-enriched mononuclear cells using the Trizol Reagent (Invitrogen Corp., Carlsbad, USA). The integrity of RNA samples was determined on agarose gels (1.2%) and spectrophotometrically, using the absorption ratio at 260/280 nm. Before the reverse transcription reaction, removal of any residual DNA contamination was done by incubating all RNA samples with RQ1-RNase free DNase I (Promega, Madison, WI, USA), at 37°C for 25 minutes, as suggested by the manufacturer.

cDNA synthesis was performed in all samples, using oligo random hexanucleotides and M-MLV (Moloney Murine Leukemia Virus) reverse transcriptase (Promega Corp. Madison, USA), as previously described [30]. The reaction volume was precipitated with NaAcetate and ethanol at –20°C overnight. The cDNAs were diluted in nanopure water and were kept as a stock at –80°C, before the real-time PCR analysis. 

### 2.4. Quantitative Real-Time PCR

A relative quantitative real-time PCR was performed in a spectrofluorometric thermal cycler (LightCycler, ROCHE, Manheim, Germany) using the LightCycler Fast Start DNA Master HybProbe kit (ROCHE, Manheim, Germany), primers and fluorescently labeled hybridization probes specifically designed by TIM-MOLBIOL (Berlin, Germany) to detect the genes of interest. The specific reaction conditions for each set of genes (primer concentration, annealing temperature, magnesium chloride) were optimized first using the SYBR Green fluorescent dye (LightCycler FastStart DNA Master SYBR Green I, Roche Diagnostics). Hybridization primers and probes for all target and reference genes were as shown in Tables 3 and 4.

Relative quantification RT-PCR is the method that determines the changes in steady-state mRNA levels of a gene across various samples and expresses them relative to the levels of an internal reference control gene, usually a housekeeping gene. In order to quantify resistin, TNF-*α*, IL-6, and IL-1*β* relative mRNA levels, we used the calibrator normalized assay with an efficiency correction and the LightCycler Relative Quantification 1.01 Software (Roche Diagnostics) as it has been described before [31]. Briefly, using this quantification method, results are expressed as the target/reference ratio of the sample divided by the target/reference ration of the calibrator, and it does not require a standard curve in each run. The human housekeeping genes of *β*-actin and porphobilinogen deaminase (PBGD) were used as standards for normalization. Selection of either *β*-actin or PBGD as housekeeping genes was necessary, because the copy number of the housekeeping gene should be in a similar range with that of the target gene to make comparative quantification possible [8]. The RNA-DNase I treated samples, in which no M-MLV (Moloney Murine Leukemia Virus) reverse transcriptase was added, together with the water containing samples (no DNA template), were used as negative controls in the RT-PCR reaction.

Relative quantification results were expressed in arbitrary units (AUs). Results from some PCR runs were run in ethidium bromide stained agarose gels and photographed in an Image Analyser VDS System (Amersham Biotech-Pharmacia, Sweden) for visualization purposes.

### 2.5. Immunoassays

Resistin (BioVendor, Czech Republic), TNF-*α*, IL-6, and IL-1*β* (Quantikine, R&D Systems, Oxon, UK) secreted proteins were measured in the supernatant from the human cultured monocyte-enriched mononuclear cells, by the use of standard commercial ELISAs according to the manufacturer recommended protocols. Circulating resistin (BioVendor, Czech Republic), TNF-*α* (R&D Systems, Oxon, UK), adiponectin, IL-6, and IL-1*β* proteins (Human Serum Adipokine Panel A and Panel B LINCOplex kits, Linco-Millipore Corp., USA) were measured in the plasma of all volunteers using either standard commercial sandwich ELISA kits or the multiplex assay (xMAP technology) and the fluorescently labeled microsphere beads in a LUMINEX 200 instrument (Luminex Corp., USA). Due to the low circulating IL-6 and IL-1*β* protein levels in the human plasma, we could not detect IL-1*β* in the plasma of the healthy individuals, while IL-6 could be detected in only 4 out of 10 individuals making the analysis of circulating IL-1*β* and IL-6 problematic. 

The sensitivities of the assays were: 0.2 ng/mL (resistin), <4.4 pg/mL (TNF-*α*-Elisa assay), <0.70 pg/mL (IL-6-Elisa assay), <1 pg/mL (IL-1*β*-Elisa assay), 0.1 pg/mL (IL-1*β*-multiplex assay), 145.4 pg/mL (adiponectin), and 1.6 pg/mL (IL-6-multiplex assay). The intra-and interassay coefficients of variation were, respectively, 3.6% and 6.7% (resistin), 4.9% and 5.8% (TNF-*α*-Elisa assay), 2.6% and 4.5% (IL-6, Elisa assay), 4.8% and 5.6% (IL-1*β*-Elisa assay), 1.4–7.9% and <21% (adiponectin, IL-6, and IL-1*β*-multiplex assays). Plasma samples were measured in duplicate in a single experiment.

### 2.6. Statistical Analysis

Statistical analysis was performed using the SPSS 14.0.1 software (SPSS Corp, Chicago, IL, USA). Data are expressed as mean ± SEM of *n* independent expreriments. Because the investigated variables showed a nonnormal distribution, nonparametric statistical analyses were applied (Spearmann's correlation test, Wilcoxon signed rank Test). Multivariate regression analysis was also performed if any adjustments were necessary. A *P* value of less than 0.05 was considered statistically significant. 

## 3. Results

### 3.1. Patients' Characteristics

The clinical and biochemical characteristics of the volunteers studied, as well as their plasma resistin, TNF-*α*, IL-6, and adiponectin levels are shown in Table 1. All individuals had normal glucose tolerance, a mean BMI of 25.7 ± 1.2 Kg/m^2^, and their CRP levels were within physiological range (0–1.4 mg/L). Circulating adiponectin levels were higher in women compared to men (30.72 ± 6.32 *μ*g/mL versus 11.26 ± 3.49 *μ*g/mL, *P* = 0.016), but no other differences were observed with regard to age, BMI, fasting insulin and glucose plasma levels, insulin resistance as assessed by HOMA-IR index, or plasma resistin, TNF-*α* and IL-6 proteins between men and women (Table 1). 

Overall, circulating adiponectin was negatively associated with HOMA-IR index (*r* = −0.636, *P* = 0.048), while circulating resistin was positively associated and plasma TNF-*α* negatively associated with FFA plasma levels (*r* = 0.773, *P* = 0.015 and *r* = −0.835, *P* = 0.005, resp.). The former significance was retained in a stepwise multivariate analysis independent of age, gender, BMI, and insulin resistance (HOMA-IR index) as independent variables (B ± SE: 10.74 ± 3.18, *P* = 0.043) (*R* ^2^ = 0.92), while the latter significance was lost in the multivariate analysis. Furthermore, circulating plasma resistin was also negatively associated with circulating plasma TNF-*α* levels (*r* = −0.678, *P* = 0.045).

### 3.2. Regulation of Resistin mRNA and Protein Expression by Insulin, Leptin, and Glucose in Human Mononuclear Cells

**Figure 1:**
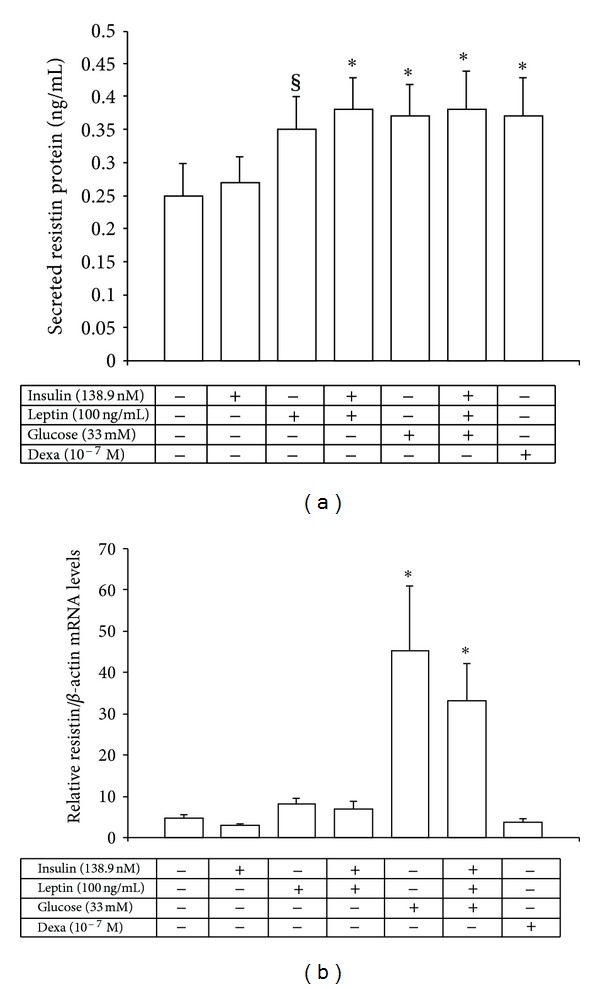
Secreted resistin protein levels (a) and relative resistin/*β*-actin mRNA levels (b) from human monocyte-enriched mononuclear cells after *in vitro* exposure for 24 h to high concentrations of insulin, leptin, glucose, and dexamethasone. *P < 0.03 versus control cells, ^§^ *P* = 0.049 versus control cells.

Leptin by itself significantly upregulated resistin protein secretion (*P* = 0.049) and tended to induce resistin mRNA levels (*P* = 0.059) from the monocyte-enriched mononuclear cells, after the 24-h incubation period, and its combination with insulin further increased by 52% resistin protein secretion (*P* = 0.024), but it did not alter basal resistin mRNA levels (Figures 1(a) and 1(b)). Insulin alone did not have any effect on resistin protein or mRNA levels. In contrast, high concentrations of glucose powerfully and significantly increased the relative resistin mRNA and protein levels (*P* = 0.004 for both), an effect that remained significant when glucose was combined with insulin and leptin (Figures 1(a) and 1(b)). Finally, dexamethasone (10^−7 ^M) increased significantly resistin protein production (*P* = 0.006, Figure 1(a)), but failed to change resistin basal mRNA levels (Figure 1(b)).

### 3.3. Regulation of TNF-*α* mRNA and Protein Expression by Insulin, Leptin, and Glucose in Human Mononuclear Cells

**Figure 2:**
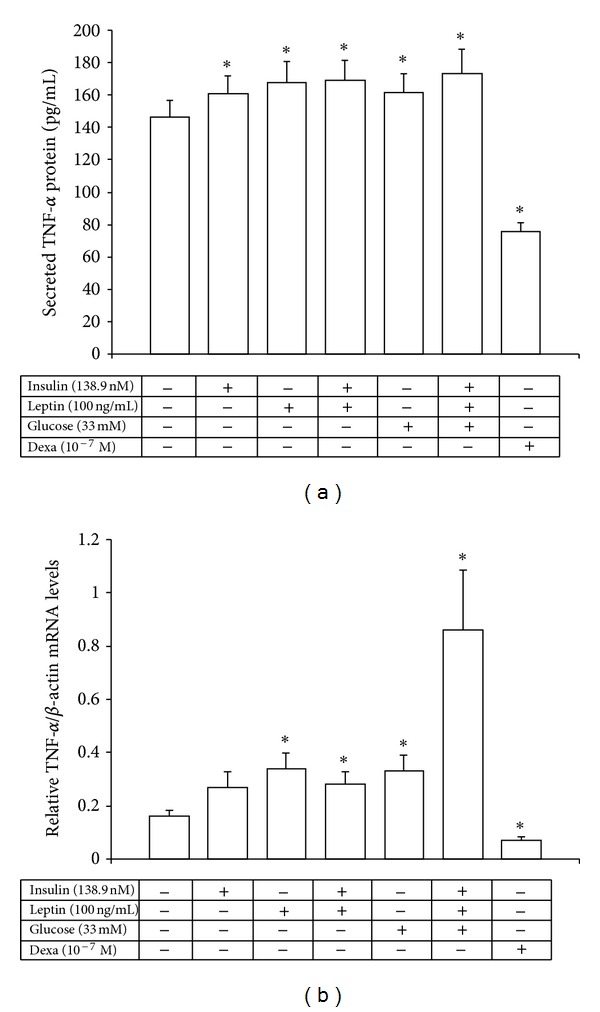
Secreted TNF-*α* protein levels (a) and relative TNF-*α*/*β*-actin mRNA levels (b) from the human monocyte-enriched mononuclear cells after *in vitro* exposure for 24 h to high concentrations of insulin, leptin, glucose, and dexamethasone. *P < 0.045 versus control cells.

High concentrations of insulin and leptin either alone or combined together significantly increased TNF-*α* protein secretion (*P* < 0.02) from human peripheral monocyte-enriched mononuclear cells *in vitro* (Figure 2(a)). Furthermore, glucose alone significantly increased TNF-*α* protein (*P* = 0.013) and its combination with insulin and leptin upregulated further TNF-*α* protein secretion levels (*P* < 0.001, Figure 2(a)). 

With regard to TNF-*α* mRNA production glucose and leptin, either alone or combined with insulin, significantly increased relative TNF-*α* mRNA levels (2.0-fold, 2.1-fold, and 5.2-fold, resp., *P* < 0.05, *P* < 0.02 and *P* < 0.002, resp.), while dexamethasone significantly suppressed by 50% both TNF-*α* mRNA levels and protein secretion *in vitro* (*P* < 0.02 and *P* < 0.001, resp.) (Figures 2(a) and 2(b)).

### 3.4. Regulation of IL-6 and IL-1*β* mRNA and Protein Expression by Insulin, Leptin, and Glucose from the Human Mononuclear Cells

**Figure 3:**
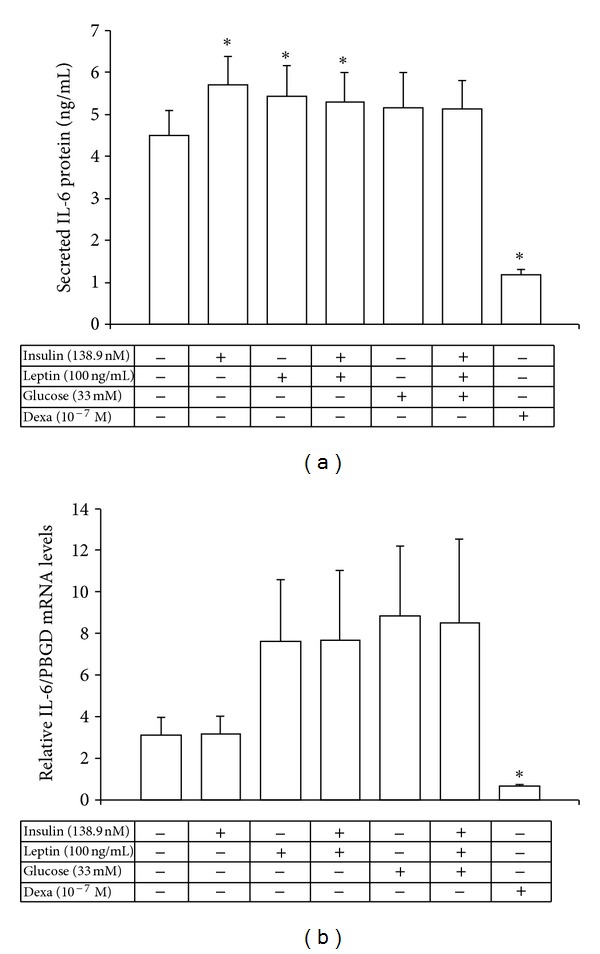
Secreted IL-6 protein levels (a) and relative IL-6/PBGD mRNA levels (b) from the human monocyte-enriched mononuclear cells after exposure to high concentrations of insulin, leptin, glucose, and dexamethasone. *P < 0.05 versus control cells.

Insulin and leptin either alone or both combined together increased significantly IL-6 protein levels (*P* = 0.005, *P* = 0.004, and *P* = 0.016, resp.) from human peripheral monocyte-enriched mononuclear cells (Figure 3(a)), while they did not change basal IL-6 mRNA levels (Figure 3(b)). Dexamethasone, however, powerfully suppressed up to 6-fold IL-6 protein and mRNA levels (*P* < 0.001 and, *P* < 0.05 resp.) (Figures 3(a) and 3(b)). Glucose on the other hand, either alone or combined together with insulin and leptin had no significant effect on IL-6 mRNA or protein secretion levels, although there was a nonsignificant 3-fold increase of IL-6 mRNA levels under these conditions (Figures 3(a) and 3(b)).

**Figure 4:**
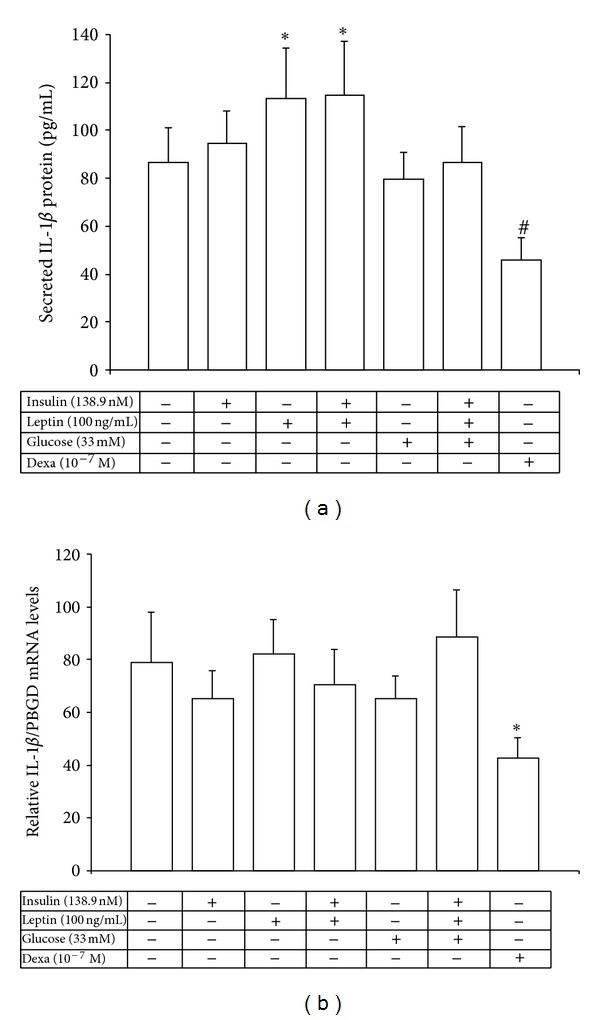
Secreted IL-1*β* protein levels (a) and relative IL-1*β*/PBGD mRNA levels (b) from the human monocyte-enriched mononuclear cells after exposure to high concentrations of insulin, leptin, glucose, and dexamethasone. *P < 0.05 versus control cells, ^#^ *P* = 0.061 versus control cells.

Only leptin or its combination with insulin had a significant increased effect on IL-1*β* protein secretion (*P* < 0.05 and *P* < 0.02, resp.) from human peripheral monocyte-enriched mononuclear cells *in vitro* (Figure 4(a)). Glucose either alone or combined together with insulin and leptin had no effect on IL-1*β* protein levels. No effect on IL-1*β* mRNA levels was observed upon incubation of the cells with glucose, leptin, insulin, or their combination (Figure 4(b)). Only dexamathasone reduced IL-1*β* mRNA (*P* = 0.013) and tended to reduce IL-1*β* protein expression (*P* = 0.061) from the human cells *in vitro* (Figure 4).

Additionally, secreted TNF-*α* protein levels correlated positively with secreted IL-1*β* protein levels (*r* = 0.733, *P* = 0.025) and TNF-*α* mRNA levels (*r* = 0.667, *P* = 0.05), while secreted IL-1*β* protein correlated negatively with CRP (*r* = −0.730, *P* = 0.025) and with TNF-*α* mRNA levels (*r* = 0.833, *P* = 0.010) (Table 2). Moreover, IL-6 basal mRNA levels correlated positively with fasting glucose plasma levels (*r* = 0.786, *P* = 0.021).

## 4. Discussion

We have demonstrated that the combined exposure of human peripheral monocyte-enriched mononuclear cells to high concentrations of insulin and leptin for 24 hours *in vitro* clearly stimulated resistin, TNF-*α*, IL-6, and IL-1*β* protein expression. The further addition of glucose increased significantly resistin and TNF-*α* protein and mRNA levels but failed to induce similar changes in IL-6 and IL-1*β* protein production. Moreover, as expected, exposure to dexamethasone decreased powerfully TNF-*α*, IL-6, and IL-1*β* production while it increased resistin protein secretion. 

The above findings are in agreement with previous results where dexamethasone or high glucose concentrations increased the expression of both resistin mRNA and protein in 3T3-L1 murine adipocytes [32]. High glucose concentrations also upregulated resistin gene expression and protein levels in the human U937 monocytic cell line, an effect mediated by MAPK and NF-kB-dependent mechanisms [33]. In contrast, insulin diminished resistin expression by 30–37% in 3T3-L1 mouse adipocytes acting possibly through the Phosphoinositide (PI)-3, ERK, or p38-MAP- kinases pathways [32, 34]. Insulin in diabetic mice caused a marked increase in adipose tissue resistin mRNA levels [35] and induced resistin protein secretion but not mRNA production in human differentiated adipocytes [36]. However, in humans, there are a number of studies that failed to identify any correlation of resistin with various markers of insulin resistance [11, 12]. In agreement with this, resistin plasma levels and gene expression in mononuclear cells and in macrophages did not differ between women with polycystic ovary syndrome (PCOS) and controls [37]. Furthermore leptin treatment either did not alter resistin mRNA expression in human peripheral blood mononuclear cells [38] or decreased adipose tissue resistin gene expression and improved insulin sensitivity in the ob/ob mice [39]. These discrepancies are probably due to the fact that resistin's expression is regulated differently between tissues as well as between mouse and human species. It is of interest that although the human resistin gene appears to be the true ortholog of the murine resistin gene, the derived proteins not only have a very low degree of amino acid conservation, but also exhibit a different tissue distribution profile [40, 41]. These findings, along with the evidence that only two RELM genes have been discovered in humans compared to three in mice, imply that the two genes possibly serve different biological functions in the two species. In our study, however, exposure of the human monocyte-enriched mononuclear cells to either dexamethasone or leptin *in vitro* induced resistin protein secretion while no change was observed in resistin mRNA levels.

We have also demonstrated that high concentrations of either insulin or leptin could enhance cytokine (TNF-*α*, IL-6, IL-1*β*) production while the addition of high glucose induced further TNF-*α* mRNA and protein expression, a finding also supported by previous reports in both humans and mice [42, 43]. Interestingly, we have previously demonstrated that monocyte-enriched mononuclear cells from individuals with type 2 diabetes show increased levels of visfatin, resistin, TNF-*α*, IL-6, and IL-1*β* mRNA expression indicating that hyperinsulinemia and hyperglycemia could enhance cytokine production [8, 31]. Surprisingly, in the present *in vitro* study we could not detect an increase in IL-6 or IL-1*β* production from the monocyte-enriched mononuclear cells isolated from healthy individuals after glucose, insulin, and leptin treatment. Notably IL-6 mRNA levels were increased around 3-fold, but not significantly, under these conditions. Finally, the inhibitory effect of dexamethasone on IL-6, TNF-*α*, and IL-1*β* production demonstrated in our study (a finding that had also been reported previously by Bessler et al. [44]), confirms that cytokine production by the isolated peripheral monocyte-enriched mononuclear cells under the *in vitro* conditions we used is subject to regulation. This serves also as a control for the changes we observed after exposure to glucose, insulin, and leptin. 

Hyperinsulinemia, hyperglycemia, and hyperleptinemia are commonly seen in obesity-related cardiometabolic disorders, like type 2 diabetes and its complications such as dyslipidemia, hypertension, and atherosclerosis. Low grade systemic inflammation seems to be the common soil hypothesis for these metabolic disorders and recently it was demonstrated that inflammasome components are key players in the induction of obesity and insulin resistance in mice [45]. Circulating monocytes as well as macrophages infiltrating the arterial wall and the adipose tissue are an important source of inflammatory cytokines, and adipokines like resistin, participating in the process of atherogenesis and contributing to systemic inflammation in the adipose tissue and the atheromas, could aggravate this process under hyperglycemic, hyperinsulinemic and possibly hyperleptinemic conditions [3, 46]. Indeed, resistin was found to be more highly expressed in atherosclerotic aneurysms than in normal arteries, while at the same time induces the proliferation and migration of the human vascular smooth muscle cells [21] leading to progression of atherosclerosis in rabbit carotid artery [47]. Plasma resistin levels were significantly higher in patients with coronary artery disease (CAD) and in individuals with acute inflammatory disease than controls, and correlated with markers of inflammation predicting coronary atherosclerosis in humans [18, 22, 23, 48]. Resistin may also induce endothelial dysfunction [19, 20, 49] and treatment of macrophages with resistin could induce lipid accumulation, supporting further resistin's role in atherosclerosis [50]. It is intriguing that in our study dexamethasone, a known anti-inflammatory agent, increased resistin protein levels in human mononuclear cells while at the same time it decreased powerfully TNF-*α*, IL-6, and IL-1*β* production. Moreover, monocytes from DM2 patients compared to controls express higher resistin mRNA levels with a similar increase in their corresponding plasma levels [8]. Resistin's proinflammatory nature is characterized by its induction by various proinflammatory molecules, while at the same time it can stimulate the expression of cytokines such as TNF-a and IL-12 [15–17]. Moroever, in inflammasome, deficient animals which are protected against the development of high-fat diet (HFD), induced obesity and insulin resistance, the production of protein resistin is significantly reduced [45]. Taken all together the aforementioned, suggest that resistin may have a major role in inflammation-associated cardiometabolic disorders [17, 51].

## 5. Conclusions

In the present study, we have demonstrated for the first time that insulin and leptin and possibly glucose, at concentrations commonly seen in obesity and type 2 diabetes powerfully stimulate resistin and cytokine proinflammatory expression in cultured human monocytes which in turn could aggravate the already increased inflammatory load. It may be suggested that with expanding obesity the increase of resistin and TNF-*α* and secondarily of IL-6 and IL-1b expression from monocytes/macrophages that infiltrate the stroma of the adipose tissue and/or the vascular endothelium could enhance the effects of their adipocyte-derived levels, and thus may contribute to the risk of atherosclerosis that accompanies obesity and type 2 diabetes.

## Figures and Tables

**Table 1:** Anthropometic and biochemical characteristics and plasma resistin, TNF-*α*, IL-6, and adiponectin levels of the 10 healthy volunteers as a whole, and further divided in women and men.

	Total subjects (*n* = 10)	Women (*n* = 5)	Men (*n* = 5)	*P* value
Age (years)	34.7 ± 1.17	32.8 ± 1.31	36.6 ± 1.6	0.056
BMI (Kg/m^2^)	25.7 ± 1.19	24.48 ± 2.28	26.83 ± 0.69	0.151
Fasting insulin (*μ*U/mL)	10.8 ± 1.3	10.38 ± 1.81	11.23 ± 2.07	0.841
Fasting glucose (mg/dL)	79.4 ± 3.67	81.96 ± 7.3	76.84 ± 1.97	0.222
HOMA-IR index	2.16 ± 0.32	2.17 ± 0.54	2.15 ± 0.41	0.690
FFA (mM)	0.22 ± 0.05	0.28 ± 0.09	0.14 ± 0.04	0.190
CRP (mg/L)	0.38 ± 0.15	0.31 ± 0.16	0.46 ± 0.31	0.905
Resistin (ng/mL)	6.27 ± 0.49	6.85 ± 0.77	5.55 ± 0.36	0.190
TN-*α* (pg/mL)	1.46 ± 0.37	1.19 ± 0.29	1.73 ± 0.69	0.841
IL-6 (pg/mL)	7.3 ± 3.90	1.69 ± 0.19	12.90 ± 5.32	0.333
Adiponectin (*μ*g/mL)	20.99 ± 4.7	30.72 ± 6.32	11.26 ± 3.49	**0.016**

BMI: Body Mass Index; HOMA-IR: homeostasis model assessment; FFA: free fatty acids; CRP: C-reactive protein. Data were expressed as means ± SEM. *P* value gives the statistical difference between women and men.

**Table 2:** Bivariate correlations of secreted resistin, TNF-*α*, IL-6, and IL-1*β* protein with their corresponding basal mRNA levels and the studied metabolic parameters.

	Secreted resistin (ng/mL)	Secreted TNF-*α* (pg/mL)	Secreted IL-6 (pg/mL)	Secreted IL-1*β* (pg/mL)
Secreted TNF-*α* (pg/mL)	−0.167			
Secreted IL-6 (pg/mL)	−0.600	0.491		
Secreted IL-1*β* (pg/mL)	0.048	0.733*	0.017	
Relative resistin mRNA levels	0.083	−0.224	−0.321	0.167
Relative TNF-*α* mRNA levels	0.143	0.667**	0.033	0.833*
Relative IL-6 mRNA levels	−0.071	−0.357	0.286	0.690
Relative IL-1*β* mRNA levels	0.071	0.381	0.429	0.036
BMI (Kg/m^2^)	−0.017	−0.127	0.042	−0.433
Fasting insulin (*μ*U/mL)	−0.317	−0.127	0.176	−0.417
Fasting glucose (mg/dL)	0.450	−0.552	−0.018	−0.517
FFA (mM)	−0.108	−0.017	0.487	−0.143
CRP (mg/L)	−0.514	−0.479	0.297	−0.730*

BMI: Body Mass Index; FFA: free fatty acids. CRP: C-reactive protein. Numbers represent *r* values. *P < 0.025, **P < 0.05.

**Table 3:** 

	5′-sense primer-3′	5′-a-sense primer-3′
Resistin	GGGCTGTTGGTGTCTAGCAAG	GTCTCGGCGCGCACAT
TNF-*α*	ACAAGCCTGTAGCCATGTT	AAAGTAGACCTGCCCAGACT
IL-6	CCAATCTGGATTCAATGAGGAGACT	GAGCCCTCAGGCTGGACTG
IL-1*β*	CAGGGACAGGATATGGAGCAA	GCAGACTCAAATTCCAGCTTGTTA
*β*-actin	CTTCTACAATGAGCTGCGTGTG	GTGAGGATCTTCATGAGGTAGTCAGTC
PBGD	GGCTGCAACGGCGGAA	CCTGTGGTGGACATAGCAATGATT

**Table 4:** 

	5′-FL probe-3′	5′-LC Red640 probe-3′
Resistin	GCGACCTCCTGGATCCTCTCATTGA	GCTTCTTCCATGGAGCACAGGGTC
TNF-*α*	GCATTGGCCCGGCGGTTC	CCACTGGAGCTGCCCCTCAGCT
IL-6	AGATGCAATAACCACCCCTGACCCAA	CACAAATGCCAGCCTGCTGACGAA
IL-1*β*	GCTTATCATCTTTCAACACGCAGGACA	GTACAGATTCTTTTCCTTGAGGCCCA
*β*-actin	GGTATGCCCTCCCCCATGCC	TCCTGCGTCTGGACCTGGCTG
PBGD	CATACAGACGGACAGTGTGGTGGCAAC	TGAAAGCCTCGTACCCTGGCCTG
